# Precision and neuronal dynamics in the human posterior parietal cortex during evidence accumulation

**DOI:** 10.1016/j.neuroimage.2014.12.015

**Published:** 2015-02-15

**Authors:** Thomas H.B. FitzGerald, Rosalyn J. Moran, Karl J. Friston, Raymond J. Dolan

**Affiliations:** a Wellcome Trust Centre for Neuroimaging, London WC1N 3BG, UK; b Virginia Tech Carilion Research Institute, Roanoke, VA, USA; c Bradley Department of Electrical & Computer Engineering, Roanoke, VA, USA

## Abstract

Primate studies show slow ramping activity in posterior parietal cortex (PPC) neurons during perceptual decision-making. These findings have inspired a rich theoretical literature to account for this activity. These accounts are largely unrelated to Bayesian theories of perception and predictive coding, a related formulation of perceptual inference in the cortical hierarchy. Here, we tested a key prediction of such hierarchical inference, namely that the estimated precision (reliability) of information ascending the cortical hierarchy plays a key role in determining both the speed of decision-making and the rate of increase of PPC activity. Using dynamic causal modelling of magnetoencephalographic (MEG) evoked responses, recorded during a simple perceptual decision-making task, we recover ramping-activity from an anatomically and functionally plausible network of regions, including early visual cortex, the middle temporal area (MT) and PPC. Precision, as reflected by the gain on pyramidal cell activity, was strongly correlated with both the speed of decision making and the slope of PPC ramping activity. Our findings indicate that the dynamics of neuronal activity in the human PPC during perceptual decision-making recapitulate those observed in the macaque, and in so doing we link observations from primate electrophysiology and human choice behaviour. Moreover, the synaptic gain control modulating these dynamics is consistent with predictive coding formulations of evidence accumulation.

## Highlights

• MEG and DCM used to characterise neuronal dynamics during decision making.
• DCM suggested plausible hierarchical network architecture.
• Rate of accumulation best explained by pyramidal cell gain.
• Results support predictive coding models of evidence accumulation.

## Introduction

Although perceptual judgements can be made rapidly, many involve integrating information over an extended period of time. Slow ramping activity within neurons in posterior parietal cortex is critical for such decisions, often interpreted as representing a gradual accumulation of evidence for one option or other (Roitman and Shadlen, 2002; Huk and Shadlen, 2005; Hanks et al., 2006; Gold and Shadlen, 2007). However, the relationship between hypothesised evidence accumulation processes and more general theories of perception has not been widely explored, it is now increasingly accepted that classical evidence accumulation schemes are a special case of generic Bayesian inference about the causes of sensory input (Bitzer et al., 2014). In this paper, we consider evidence accumulation as perceptual inference in the context of predictive coding and ask whether we can understand the neuronal correlates in this light.

Predictive coding is an influential theory of perception (Rao and Ballard, 1999; Friston, 2008; Summerfield and Egner, 2009) and brain function (Friston, 2010) in which inference is realised in message passing between hierarchically organised brain regions (Felleman and Van Essen, 1991; Lee and Mumford, 2003; Friston, 2008), with top-down signals encoding predictions and bottom-up signals encoding prediction errors. In brief, ascending prediction errors are accumulated by high-level units encoding posterior expectations. In order to approximate Bayes-optimal inference, the brain needs to represent the estimated precision (inverse variance) of ascending sensory signals (prediction errors) (Feldman and Friston, 2010). The expected precision is proposed to play a key role in weighting sensory evidence against prior beliefs and optimises the rate of evidence (prediction error) accumulation.

**Fig. 1:**
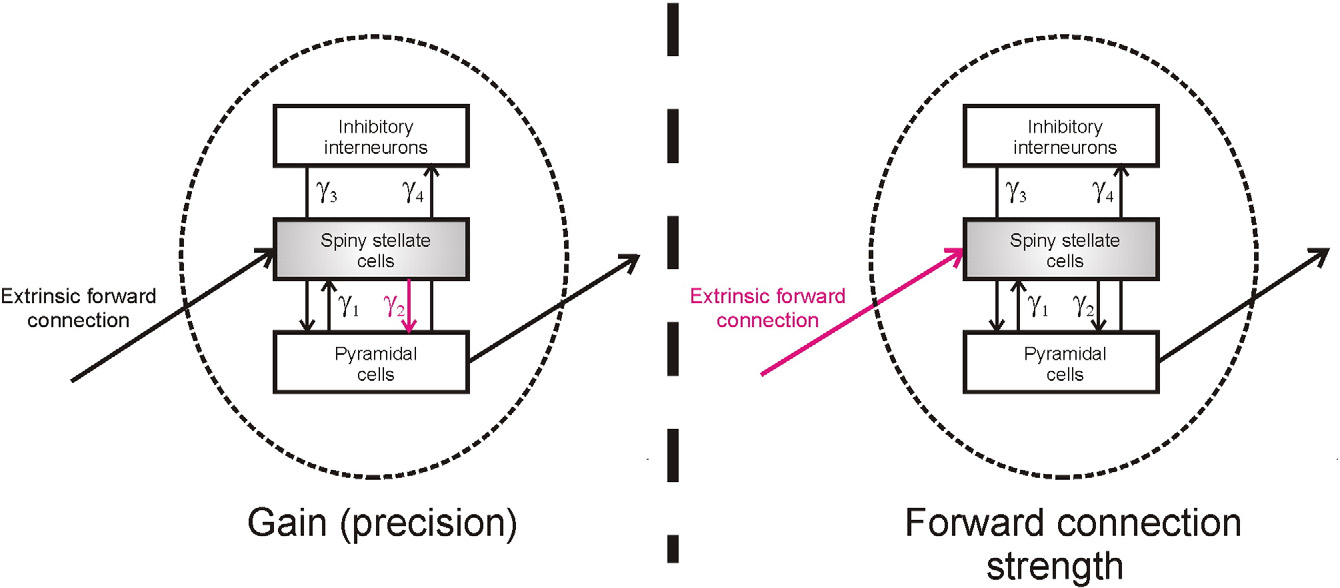
Illustration of candidate neuronal mechanisms underlying behavioural and neuronal response time variability. Our model contains three neuronal populations, input receiving spiny stellate cells, pyramidal cells that send extrinsic forward connections to other cortical regions, and inhibitory interneurons (Jansen and Rit, 1995; David and Friston, 2003). The strength (gain) of connections between these populations is parameterised by *γ*_1 − 4_. Based on predictive coding, we hypothesised that response time variability would be driven by changes in the precision of ascending sensory information, which is operationalised in our model as the gain (*γ*_2_) on the connection between input receiving stellate cells and the pyramidal cell population (left). We compared models in which the strength of intrinsic gain changed with response time, compared to changes in extrinsic forward connections between regions (right), and a null model in which response time had no effect (not shown).

In the context of evidence accumulation, a natural hypothesis is that precise sensory information should produce faster responding, via an increased ramping of neuronal activity in PPC. It has been proposed that precision is encoded by a gain of (prediction-error signalling) superficial pyramidal cells (Friston and Kiebel, 2009; Bastos et al., 2012; Brown and Friston, 2012), a hypothesis supported by biophysically plausible modelling of electrophysiological data recorded during perceptual tasks under conditions of varying sensory precision (Garrido et al., 2009; Brown and Friston, 2012; Moran et al., 2013). Here, we measured in vivo activity using source localised MEG and tested an hypothesis that the speed of subject's responses on a simple perceptual decision-making task would vary with the gain on pyramidal cells in the network of regions supporting decision-making as estimated by dynamic causal modelling (DCM), using validated and biophysically plausible models (Jansen and Rit, 1995; Daunizeau et al., 2011). In the context of the model, pyramidal cell gain is captured by modulating the strength of connections between spiny stellate and pyramidal cells, as this directly alters the sensitivity of pyramidal cell responses to extrinsic inputs (see Fig. 1). We compared this against a hypothesis that response speed would vary with the strength of extrinsic forward connections, which only indirectly alters pyramidal cell activity, and does not change the responsiveness of pyramidal cells to a given input from stellate cells. Note that there is an intimate relationship between the gain or sensitivity of pyramidal cells and the speed of their responses to (or accumulation of) presynaptic input. This is because the (intrinsic) connectivity modelling gain plays the role of a synaptic rate constant.

Depending upon both the nature of the perceptual task and the way in which sensory precision is manipulated, one might expect to find changes in pyramidal cell gain at different levels of the cortical hierarchy. Here, given that sensory precision is modulated by varying the motion coherence (rather than the properties of individual dots or their motion), gain modulation in regions encoding hypotheses about motion direction (putatively the PPC) is likely to be key. Nonetheless, it is conceivable that this might also be accompanied by precision changes in other areas. Formally speaking, the key determinant of accumulation dynamics rests upon the precision of ascending prediction errors, relative to the precision of prediction errors at the hierarchical level in question. This means that there is likely to be a delicate and balanced gain control at all levels of the cortical hierarchy. This notion is consistent with (the multilevel) projections that might mediate precision or gain control (e.g., thalamocortical projections from the pulvinar).

Using DCM, a biologically grounded form of spatiotemporal source reconstruction, we were able to ask whether activity in human PPC resembles that recorded in monkeys. Because MEG sums activity across millions of neurons, we cannot test for slow ramping activity in decision-selective cells, as typically reported in primate single-unit studies (Gold and Shadlen, 2007). Instead we need to derive predictions about population-level activity. Previous work considering population level activity in mutual and pooled inhibition models of decision-making suggests that over the course of a trial activity in the PPC should gradually increase, and that the rate of increase will correlate with response speed (Wang, 2002; Bogacz et al., 2006; Wong and Wang, 2006; Hunt et al., 2012). Our hypothesis was that precision encoding (the gain of superficial pyramidal cells) is the mechanism underlying these responses.

## Methods

### Subjects

Twenty one (13 female) right handed subjects, age range 19–29, participated in the study. All subjects were free of neurological or psychiatric disease and provided informed consent. The study was approved by the Joint National Hospital for Neurology and Neurosurgery (University College London Hospitals NHS trust) and Institute of Neurology (University College London) Ethics Committee.

### Stimuli & task

**Fig. 2:**
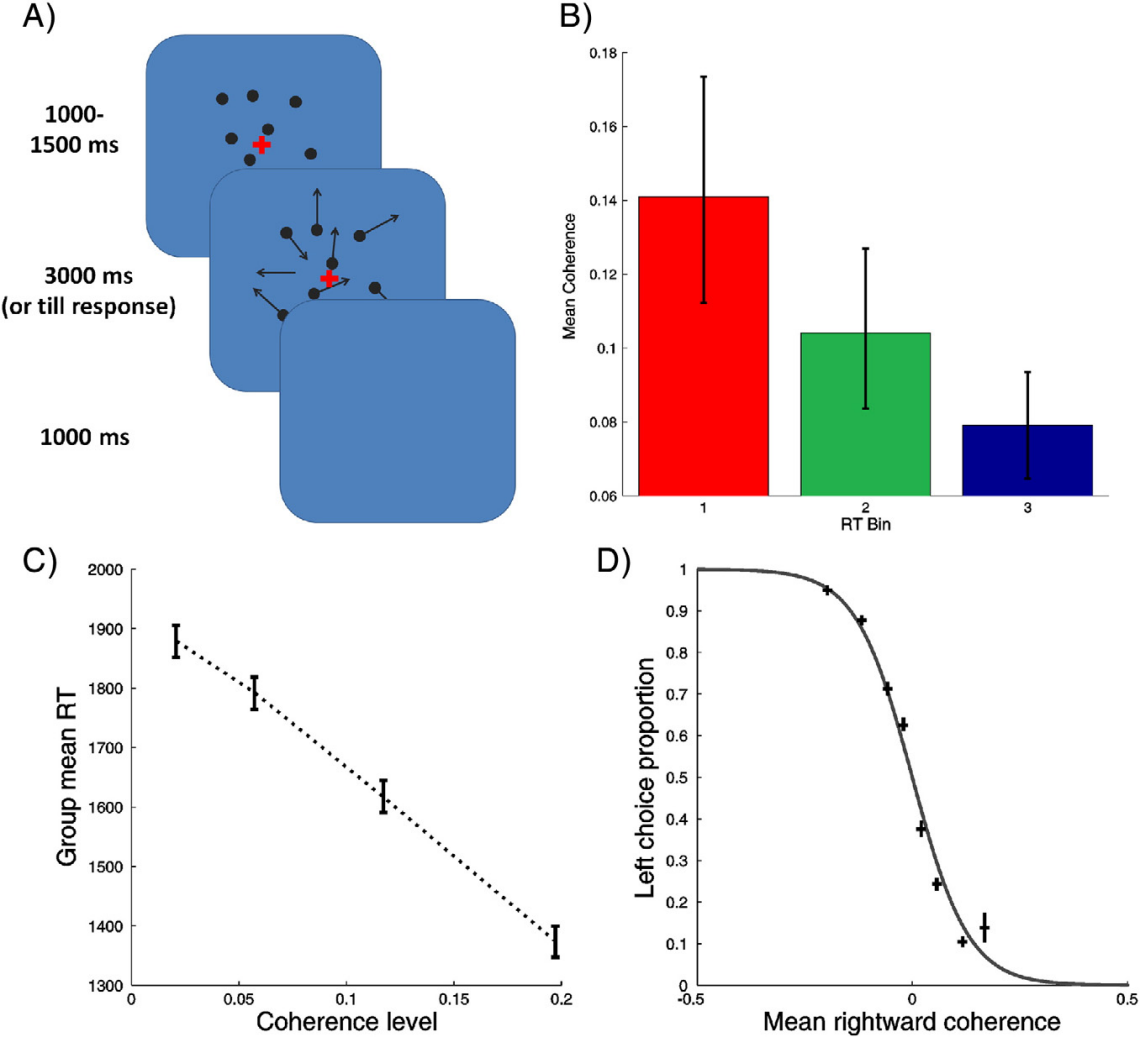
A. Top left panel: Illustration of a single trial of the direction discrimination task. A red fixation cross appeared, surrounded by a field of stationary dots. After an interval, jittered between 1000 and 1500 ms, the dots began to move. A subset of the dots moved coherently, and the rest moved randomly. Subjects had 3000 ms to make their decision; indicating whether they thought the direction of coherent motion was to the left or to the right. Dots disappeared from the screen once the decision was made, and a blank screen was shown for the remainder of 3000 ms, plus a further 1000 ms. B. Top right panel: Bar plot showing the mean coherence level of trials in each of the three reaction time bins used to analyse the behavioural data. Reaction time and motion coherence level showed a strong negative correlation. (Red: RT bin 1 (fastest). Green: RT bin 2 (intermediate). Blue: RT bin 3 (slowest)) (error bars indicate bootstrapped 95% confidence intervals). C. Bottom left panel: Average reaction time for each coherence level averaged across subjects. Reaction times showed a strong negative relationship with coherence level. (Error bars indicate bootstrapped 95% confidence intervals). D. Bottom right panel: Choice behaviour averaged across subjects. Both direction of motion and coherence level strongly affected choice behaviour, which was well fit with a sigmoid function. (Error bars indicate bootstrapped 95% confidence intervals).

Subjects performed a simple direction-discrimination task on a field of moving dots (Fig. 2). Uncertainty (or precision) was varied by changing the proportion of dots that were moving coherently in the target direction (either right or left), versus those moving randomly. 185 dots, moving at approximately 10.7 degrees per second, were presented in a circular field subtending a radius of approximately 10°. Subjects responded using two keypads, one held in either hand, and were free to respond as soon as they wished.

In each trial, a red cross (which subjects were instructed to fixate on) appeared in the middle of the screen, surrounded by a field of stationary dots. After a delay jittered between 1000 and 1500 ms, the dots started moving. Motion continued for 3000 ms, or until the subject pressed a response key. Once the dots had disappeared, a blank screen appeared for 3000 ms minus the time subjects had taken to respond (to ensure trials were evenly spaced), followed by an inter-trial interval of 1500 ms.

Subjects first performed a 112 trial practise session to calibrate task performance. Responses were then fit with a logistic function to derive individually tailored coherence levels that provided four coherence levels, corresponding to performance levels of approximately 55, 70, 85 and 95% correct. These coherence levels were held constant throughout the main experiment. During recordings, subjects performed three sessions of the task, each comprised of 112 trials. Four coherence levels were used for each direction of motion, giving eight trial types in all, presented randomly within each session.

### Behavioural analysis

To test an effect of coherence level on response accuracy we removed trials on which subjects failed to respond, and then performed a logistic regression on response data from each subject. Group level statistics were performed on the regression coefficients using a nonparametric Wilcoxon signed rank test.

To examine the predicted relationship between RT and coherence level, for each subject we categorised trials into three reaction time bins (slow, middle and fast) and analysed the mean coherence levels in each bin across subjects. Three pair wise comparisons between bins were performed using a nonparametric Wilcoxon signed rank test.

### MEG data acquisition and preprocessing

MEG data were acquired using a CTF 275-channel whole-head MEG system at a sampling rate of 600 Hz. To allow continuous monitoring of subjects' head positions, three energised electrical coils were attached to the fiducials (left and right pre-auricular, nasion). Data were downsampled to 200 Hz, bandpass filtered between 0.1 and 40 Hz and epoched between 1000 ms before movement onset to 3000 ms after. Independent component analysis was applied and components corresponding to eye movements and physiological noise were discarded. We then performed a further visual inspection on the data, and excluded any trials that still contained artefacts. Finally, we baseline corrected to the average baseline between 0 and − 200 ms. All preprocessing was performed in SPM8 (Wellcome Trust Centre for Neuroimaging, London, UK, www.fil.ion.ucl.ac.uk/spm), except for independent component analysis, which was performed using Fieldtrip (Oostenveld et al., 2011).

For each subject, trials on which they either made errors or failed to respond were excluded, and the remainder divided up into three equal-sized bins according to reaction time. These were then averaged, giving three evoked responses per subject. We adopted this binning strategy, rather than treating reaction times as a parametric regressor across trials, for computational expediency and statistical efficiency. Although a fine-grained binning can reproduce parametric analyses, it entails inverting many more (potentially noisier) event related fields.

### MEG source reconstruction

To identify areas generating these ERFs, we performed source reconstruction using Multiple Sparse Priors (MSP) (Friston et al., 2008) on the grand average evoked responses across all subjects, over the interval 0–500 ms post motion onset. We selected this interval as it enables an efficient estimation of ramping that is not confounded by activity related directly to motor responding, since for all RT bins subjects responded more than 500 ms after the end of the time window of interest (Fig. 1). That this strategy was successful is indicated in our source localisation analysis, which suggests that the main contributors to activity between 0 and 500 ms after stimulus onset were occipital and parietal responses, rather than frontal (we do not wish to make any strong claim about whether ramping activity in the posterior parietal cortex itself reflects ‘pure’ perceptual decision-making, or rather an unfolding of decisions in action space (Cisek, 2007), which falls beyond the scope of this study). Source reconstruction and dynamic causal modelling (DCM) were performed using SPM12 (Wellcome Trust Centre for Neuroimaging, London, UK, www.fil.ion.ucl.ac.uk/spm).

The MSP source reconstruction highlighted three bilaterally symmetrical regions contributing most to the evoked response between 0 and 500 ms: namely, early visual cortex (VC), a middle temporal area (MT), and posterior parietal cortex (PPC) (Fig. 2). This corresponded closely to our predictions, since these are all regions known to be involved in processing of motion (Britten et al., 1993; Cook and Maunsell, 2002) and are also implicated in perceptual decision-making (Ditterich et al., 2003; Hanks et al., 2006; Gold and Shadlen, 2007).

### Dynamic causal modelling

Dynamic causal modelling uses a biophysically plausible neuronal mass model (of hidden neuronal dynamics), an electromagnetic forward model (which maps hidden neuronal states to observed data), and a variational Bayesian inversion scheme to make inferences about neuronal connectivity and synaptic efficacy (Kiebel et al., 2009). In the neuronal model, each cortical source comprises three neural subpopulations (Jansen and Rit, 1995; David and Friston, 2003): inhibitory cells, spiny cells, and pyramidal cells, that are coupled by differential equations based on known (intrinsic) connectivity between cortical layers (Jansen and Rit, 1995). Sources are linked by extrinsic connections, based on known connectivity rules (Felleman and Van Essen, 1991), where extrinsic forward connections are excitatory, and extrinsic backward connections are inhibitory and excitatory. In such models, the effect of precision, as posited by generalised predictive coding (Friston and Kiebel, 2009; Bastos et al., 2012) is modelled as the gain of superficial pyramidal cells. In this model, we operationalise this gain as an intrinsic connectivity parameter that amplifies bottom-up signals — from the forward input-receiving spiny stellate cells in the granular layers to the pyramidal cell population (Fig. 1). We tested this hypothesis against the alternative that changes in response time were determined by modulations in the strength of extrinsic forward connections between cortical regions.

Based on the MSP source reconstruction, which was highly consistent with our a priori predictions based on the existing literature (Gold and Shadlen, 2007), we modelled evoked responses between 0 and 500 ms post-motion onset with six sources, left and right VC ([− 19/19 − 86 − 14], MNI coordinates), left and right MT ([− 46/46 − 70 − 6], MNI coordinates), left and right PPC ([− 33/33 − 48 40], MNI coordinates) using 8 spatial modes.

Inputs were modelled as a sustained (cumulative Gaussian) input with a prior mean at 200 ms post-motion onset and a prior standard deviation of 16 ms. This input was based on single unit studies, which suggest that decision-related activity is only seen in the lateral intraparietal area after 220 ms (Roitman and Shadlen, 2002; Huk and Shadlen, 2005; Gold and Shadlen, 2007). Note that in the dynamic causal models that we use the mean and standard deviation of the input are free parameters that are optimised to fit the data. This provides a considerable degree of robustness against misspecification, and allows for inter-individual variability in the shape and timing of inputs. Additionally however, we checked the appropriateness of this prior by varying its mean between 100, 150, 200 and 250 ms (using the winning model of the grand average evoked response from the network structure selection step described below). An input with a mean at 200 ms had the highest evidence, with a log Bayes factor of 73.50 and a posterior probability of > 0.999, compared with the next best model, and a prior mean of 200 ms was thus used for all subsequent analyses.

**Fig. 3:**
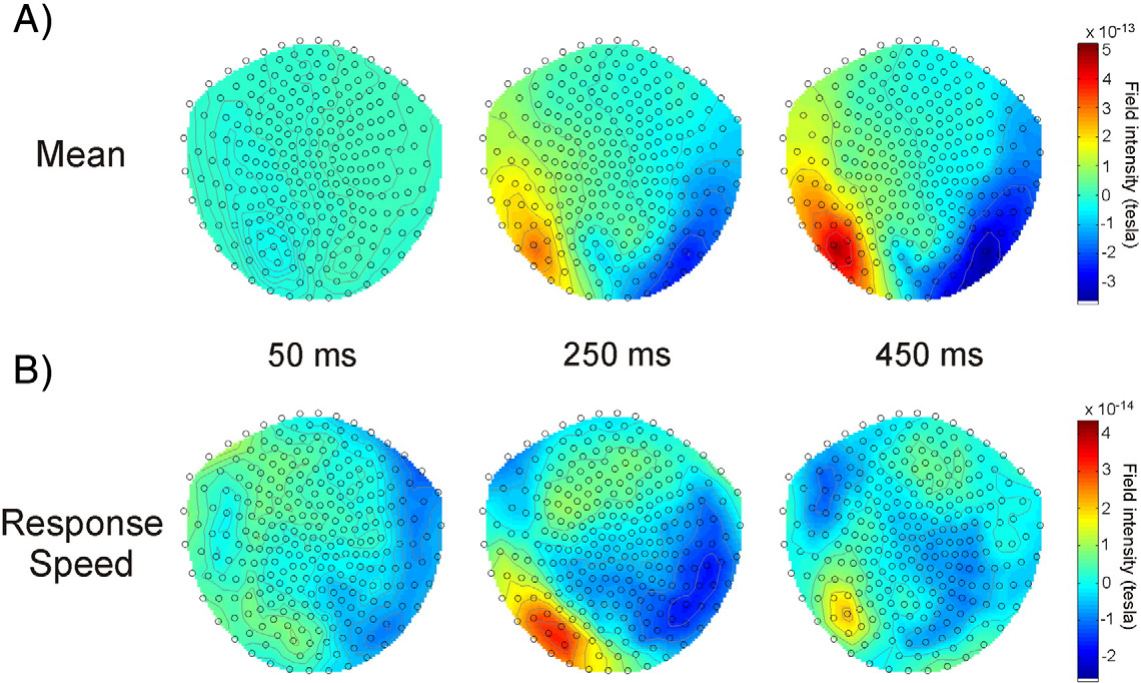
A. Top row: Topoplots illustrating grand mean evoked fields, averaged across all time bins, at 50, 250 and 450 ms. B. Bottom row: Topoplots illustrating the effect of response speed (faster responses minus slower) on grand mean evoked fields at 50, 250 and 450 ms. Response speed shows a similar topography to evoked fields averaged across conditions, consistent with its correlations with stronger (and faster rising) activity in the same cortical areas.

To establish the connectivity structure between sources, we specified 10 plausible models (Fig. 3) representing both serial and parallel hierarchies, with and without inter-hemispheric connections. These models were fitted separately to the grand average evoked response for each of the three response speeds, and fixed effects Bayesian model selection (BMS) was used to identify the best model by pooling over the three conditions.

Having established the network architecture that best modelled the data as a whole, we then characterised how network activity varied between the three reaction time conditions. Here, we use reaction time (RT) as a proxy for the underlying confidence or precision employed by the subjects. We fitted three families of models (ten in total), that differed only in how RT modulated synaptic gain, to single subject data and performed family level random effects inference (Penny et al., 2010). The first (‘gain’) family consisted of six models where RT modulated the intrinsic forward (amplifying) connection from spiny stellate to the pyramidal cells, within VC, MT, PPC or any combination of the three. The second (‘connection’) family consisted of three models in which RT modulated forward connections from VC to MT, from MT to PPC, or both (models 8–10). The third (‘null’) family comprised a single model in which RT had no effect.

To test for regionally specific effects of the RT modulation on connectivity, we performed Bayesian model averaging over the winning (‘gain’) family of models for each subject, applying Occam's window with a minimal posterior odds ratio of 0.05 (Penny et al., 2010). Group-level statistical analysis was performed on parameter estimates for the RT modulation from the winning family separately for each region averaged across hemispheres. We tested whether the RT modulation was significantly greater than zero, using a one-tailed non-parametric Wilcoxon signed rank test Bonferroni corrected for three comparisons. Interactions between the strength of effect between regions were tested using a two-tailed signed rank test Bonferroni corrected for three comparisons.

### Intrinsic variability

Our primary hypothesis concerns the neurobiological mechanisms underlying response-time variability, irrespective of whether this depends on ‘external’ (here the motion coherence level), or ‘internal’ factors. We thus varied coherence levels to induce variability between trials, and exploited this source of experimental variance when estimating model parameters. However, it is also interesting to ask whether ‘intrinsic’ variability – within each coherence level – is also explained by changes in gain on pyramidal cells. To this end, we performed an additional analysis, where we separated trials within each coherence level into three equal sized bins, and then averaged across coherence levels for each bin. Thus any differences between bins cannot be explained by coherence levels, but instead reflects intrinsic variability. We then fitted the same three families of models described above and performed random effects inference to compare the ensuing families.

### Population activity

To test our predictions about neuronal dynamics in PPC, and how it might vary at different RT levels, we reconstructed predicted pyramidal cell activity from the winning family separately for each region and each subject. We normalised activity levels for each region by the maximum estimated level, averaged across hemispheres and plotted the average responses. For each subject, predicted activity was estimated by taking an average of the responses predicted by each model in the winning family, weighted by the (normalised) posterior probability for that model and subject from the random effects analysis. This procedure is analogous to Bayesian parameter averaging (Penny et al., 2007), but applied to the responses predicted on the basis of the parameters.

### Sensitivity analysis

**Fig. 4:**
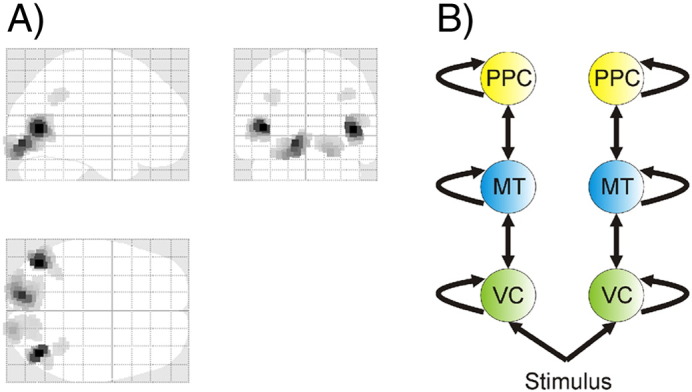
A. Left panel: Active sources from MSP reconstruction of evoked responses from 0 to 500 ms (the image shows the 200 most activated voxels). Three bilateral sources were found, in the early visual cortex (VC), the middle temporal area (MT) and the posterior parietal cortex (PPC). B*.* Right panel: Winning network structure from our dynamic causal modelling analysis. This reveals a plausible hierarchy in which PPC sits at the top, VC at the bottom, and MT in between.

To provide a qualitative illustration of the effects of varying the intrinsic gain and forward connection parameters, we performed a contribution or sensitivity analysis (the change in response with respect to the change in a parameter). Here, we simulated data using the winning network structure (Fig. 4), with all the parameters set to their Bayesian model average across subjects. We then increased the strength of the intrinsic connection within the parietal cortex by a small amount, and used the difference between the ensuing responses to assess the effects of intrinsic gain on PPC activity. To compare these effects to those generated by altering forward connections strengths, we repeated this analysis for the forward connection from MT to PPC. For graphical illustration, the sensitivity to changes in parameters was normalised to the maximum response.

## Results

### Behaviour

Within the entire data set, coherence level strongly affected both the proportion of correct trials and reaction times (Fig. 2). Coherence level and response accuracy were positively related (mean response accuracies going from high to low coherence: 91.2%, 87.3%, 74.1%, 62.0%), whilst coherence level and RTs were negatively related (mean RTs: 1880 ms, 1792 ms, 1617 ms, 1374 ms). Group level analysis of regression coefficients showed that both relationships were highly significant at the group level (median regression coefficient for coherence: 18.4, for RT: − 4580, both *p*_20_ < 0.001, Wilcoxon signed rank test). On average, subjects failed to respond on 2.5% of trials.

Dividing the data by response time (as used in the DCM analysis) showed a similar pattern; with mean reaction times for each of the three bins being 1068 ms, 1563 ms and 2186 ms respectively. As expected, average coherence level decreased as reaction time increased (Fig. 1). Mean coherence levels for each bin were 14.1% (bin 1), 10.4% (bin 2), 7.9% (bin 3) (all differences significant at *p*_20_ < 0.001, Wilcoxon signed rank test). This establishes a behavioural validation of the experimental stimulus manipulations and associates faster reaction times with more coherent (precise) sensory information.

### Source reconstruction & network selection

Source reconstruction showed three bilateral sources with the largest posterior estimates of evoked power (the sum of squared source activity during the time window of interest) (Litvak and Friston, 2008). These located to early visual cortex (VC), a mid-temporal cortical area (MT) and posterior parietal cortex (PPC) (Fig. 2). These locations represent a plausible functional architecture, given the well-established role of MT in processing motion (Britten et al., 1993; Treue and Maunsell, 1996; Chawla et al., 1998), and the critical importance of posterior parietal cortex for this type of perceptual decision-making task (Gold and Shadlen, 2007).

**Fig. 5:**
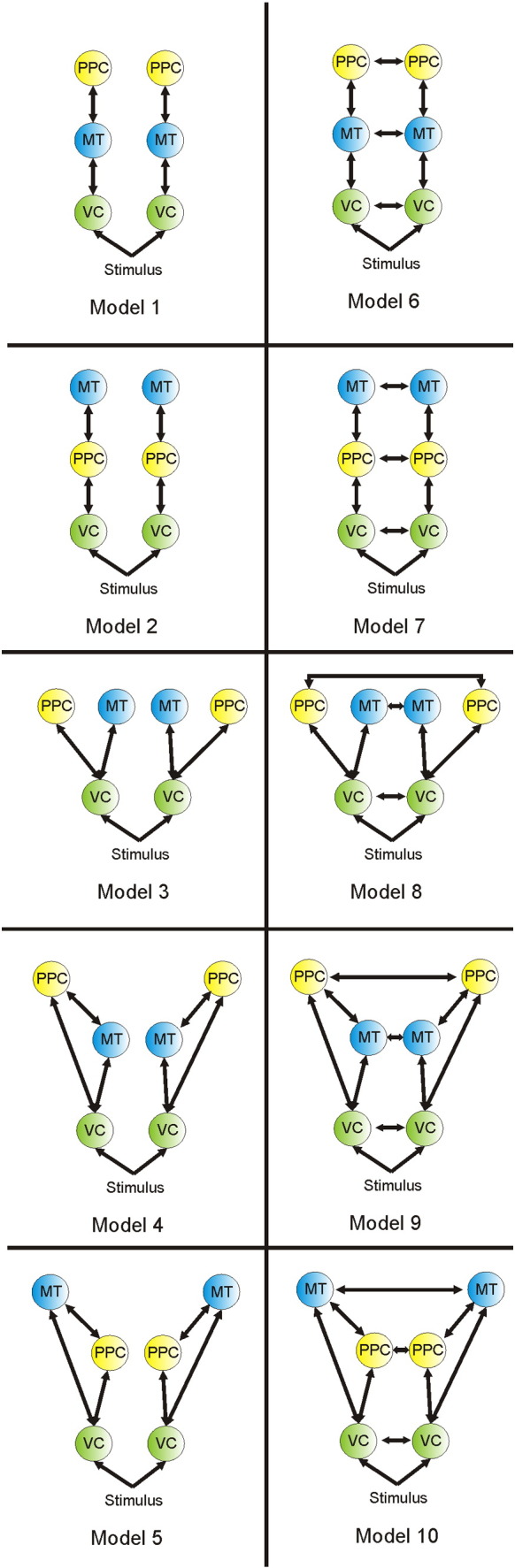
Models used for initial network selection. We tested 10 anatomically plausible models, in linear and parallel hierarchies, with and without lateral connections. Regions at the same level in the figure are connected by lateral connections; regions at different levels are connected by both forward connections (running from lower to higher levels) and backward connections (running from higher to lower).

Bayesian model selection suggested that the network structure that best described the data was a linear hierarchy, with PPC at the top, MT in the middle and VC at the bottom (Figs. 4,5). The winning model had a log Bayes factor of greater than 28, compared to the next best model (which had the same network structure, but added lateral connections) and a posterior probability of > 0.9999. This fits precisely with what one would predict, given the known anatomy of the visual system (Felleman and Van Essen, 1991).

### Estimated precision

In line with our prediction that reaction time reflected the estimated precision of sensory evidence, the results of our family level random effects inference (Penny et al., 2010) strongly favoured the ‘gain’ family of models, in which RT modulated the intrinsic amplifying connection from spiny stellate to pyramidal cells, rather than the ‘connection’ family in which it modulated the strength of ascending connections between regions, or the ‘null’ family in which it had no effect (exceedance probability 0.986).

Group level analysis of single subject parameter estimates – generated by Bayesian model averaging across the winning family – showed a significant RT modulation in the PPC (*μ* = 1.090, *p*_20_ = 0.012) but not in MT (*μ* = 0.992, *p*_20_ > 1.000) or VC (*μ* = 1.022, *p*_20_ = 0.153) (all statistics Wilcoxon signed rank test, Bonferroni corrected for three comparisons). None of the differences showed a significant interaction with region, when corrected for multiple comparisons. Thus, our findings confirm that the effect size of RT-dependent gain on pyramidal cells in the PPC is large in relation to intersubject variability but cannot preclude similar effects in MT and VC.

### Intrinsic variability

To assess whether intrinsic variability (in other words variability not induced by changes in motion coherence level) was also reflected in estimated precision, we performed family level random effects inference on data binned for each coherence level. Consistent with our main findings, the family of models in which RT modulated pyramidal cell gain was strongly favoured (exceedance probability 0.989), suggesting that a substantial component of intrinsic variability is mediated by estimated precision.

### Neuronal dynamics in the PPC

**Fig. 6:**
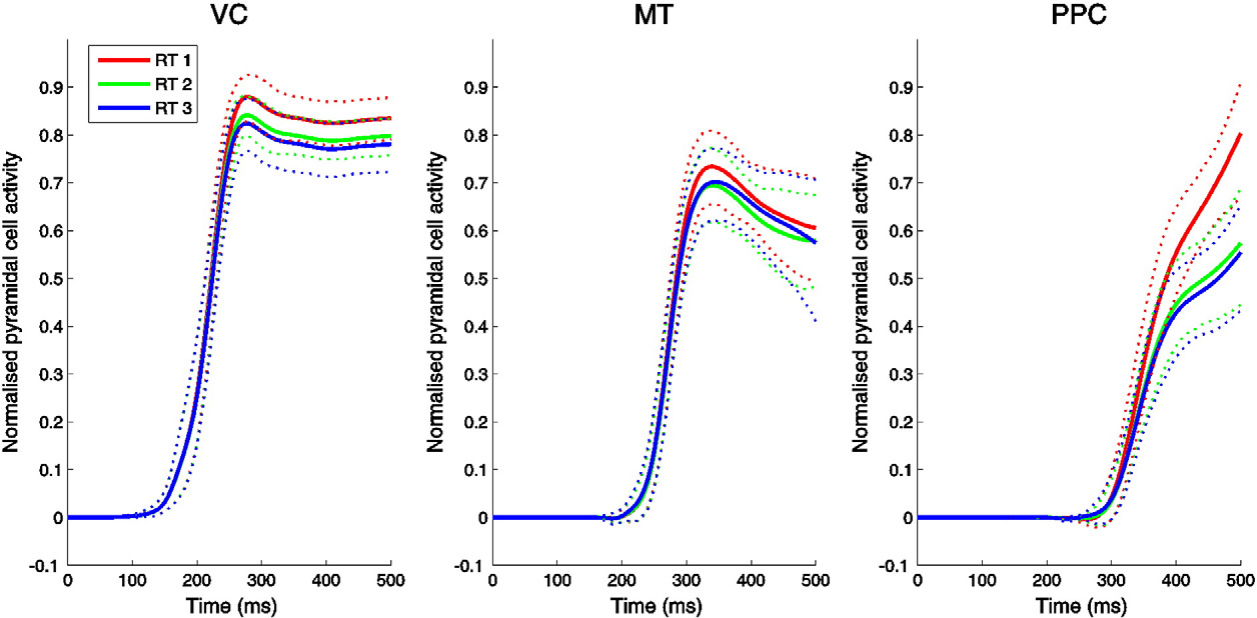
Normalised pyramidal cell activity averaged across subjects for each of the sources in the winning DCM. Posterior parietal cortex (rightmost figure) showed slow ramping activity modulated by response time, as predicted for a putative decision network, but this was not observed in any other region. (Red: RT bin 1. Green: RT bin 2. Blue: RT bin 3. Dotted lines indicate bootstrapped 95% confidence intervals).

Consistent with the idea that slow ramping activity similar to that observed in monkey PPC during perceptual-decision making occurs in humans, reconstructed pyramidal cell activity in the PPC closely matched predictions of biophysical decision-making models (Wang, 2002; Wong and Wang, 2006; Hunt et al., 2012), and closely resembled that recorded from macaque parietal cortex (Cook and Maunsell, 2002). Specifically, neuronal dynamics in the PPC source showed slowly ramping activity that was faster for lower RT trials (Fig. 6). The form of this ramping is exactly consistent with the predictions of mutual inhibition models (Bogacz et al., 2006; Wong and Wang, 2006; Hunt et al., 2012). Activity in the other sources of the DCM did not resemble that in the PPC (Fig. 6), suggesting that they do not express the same form of decision-making dynamics that were recruited by this task.

### Sensitivity analysis

**Fig. 7:**
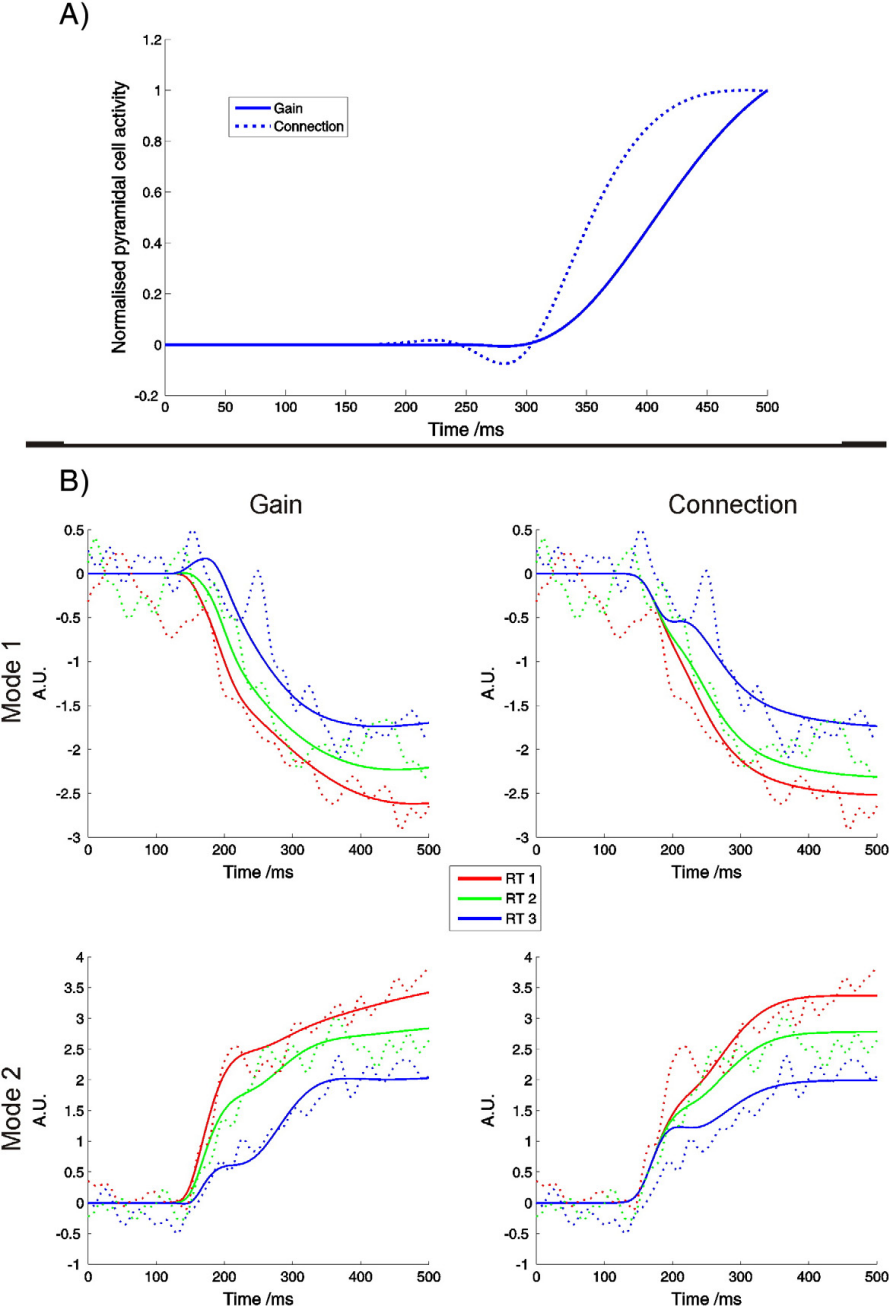
A: Top panel. Sensitivity analysis. To provide a qualitative illustration of the different predictions of gain and connectivity model families, we simulated the effect of small changes in model parameters on activity in the left PPC (all parameters other than those being perturbed were set to their posterior expectations). Altering the gain on the intrinsic connection within the PPC produced changes that increase smoothly (solid line), whereas altering the strength of the forward connection from MT to PPC produces an initial dip followed by a faster increase in activity which rapidly reaches a plateau (dotted line) (similar patterns were observed in right PPC). B: Bottom panel. Model fits. The fits of the gain (left) and forward connection (right) model families in sensor space for an illustrative subject. Responses are summarised by the first two principal spatial modes (used for data reduction — we used eight modes in total). Solid lines indicate predicted responses, dotted lines indicate observed data. The gain model family provided a significantly better fit to the data, as illustrated here by a closer correlation between predicted and observed responses (Red: RT bin 1. Green: RT bin 2. Blue: RT bin 3).

The results of our sensitivity analysis showed that changes in the gain of intrinsic connections within PPC, when compared with changes in the strength of forward connections from MT to PPC, produced clearly differentiable responses. Gain modulations produced changes that increased smoothly and approximately linearly across the time window of interest. Modulations in the strength of forward connections by contrast produced changes which, after an initial dip, increased more rapidly before reaching a plateau (Fig. 7).

## Discussion

Understanding basic mechanisms in decision-making is crucial for any account of human or animal behaviour. A rich literature on slow ramping activity in the posterior parietal cortex and its putative role in evidence accumulation (Gold and Shadlen, 2007) has developed separately from more general theories of perception and brain function (Lee and Mumford, 2003; Friston, 2010). We sought to bridge this gap by testing a key prediction of generalised predictive coding in the context of decision-making requiring integration of information over a significant period of time. In predictive coding schemes, a key role is played by the expected precision or uncertainty associated with bottom-up sensory information (prediction errors), relative to the precision of top-down predictions. The expected precision is thought to be encoded by the gain on superficial pyramidal cells that report prediction error (Friston and Kiebel, 2009; Bastos et al., 2012). In the context of perceptual evidence accumulation, this suggests that higher estimates of precision should result in faster accumulation, and thus faster responding. Evidence in support of this hypothesis thus represents an important step towards setting the literature on evidence accumulation during decision-making into a broader (normative) context.

We found that, in line with normative (Bayesian) accounts, precision (as reflected by the intrinsic connections to pyramidal cells) is strongly correlated with both the speed of decision-making and the slope of ramping activity. This extends the findings of previous studies that suggest a key role for precision in modulating both the gain on sensory signals and psychophysical performance (Garrido et al., 2009; Rahnev et al., 2011a, 2011b; Brown and Friston, 2012; Kok et al., 2012; Vossel et al., 2013) in other types of perceptual tasks. It also suggests that decisions which require integrating information over an extended period can be understood within generalised predictive coding, something we will address in future theoretical work. Our particular focus will be on the accumulation of evidence – not just for one perceptual category or other – but for the precision (reliability) of incoming sensory information. In principle, this application of generalised predictive coding has the potential to explain deviations of empirical responses from current normative accounts, like drift-diffusion models.

Although our findings support mechanistic predictions made by predictive coding, it is conceivable that other formulations could make similar predictions. Indeed, given that predictive coding is (Bayes) optimal, it would be worrying if an alternative normative formulation (such as drift or race models) could not be formulated in terms of predictive coding. Thus, all one can conclude from our results is that they are consistent with neuronally plausible (predictive coding) implementations of (Bayes optimal) evidence accumulation. This is important because, unlike other normative accounts, predictive coding also provides an explanation for a vast array of other perceptual, cognitive and motor phenomena, ranging from perceptual categorisation, through visual search to sensory attenuation during action. Having said this, to assert that the brain uses predictive coding to accumulate evidence will require multiple lines of converging evidence, which we and others are actively pursuing. Of particular note in this context is a recent study reporting that gain control during perceptual decision-making depends upon stimulus variability, exactly as a predictive coding account would predict (Cheadle et al., 2014).

Our analysis bridges a gap between our knowledge of decision circuits in non-human animals such as the macaque and humans (Gold and Shadlen, 2007). To a large extent, this issue is methodological. Decision-processes are, by nature, fast and dynamic and difficult to study with methods such as fMRI. One way to bridge this divide is to use predictions of large scale neuronal activity in the analysis of EEG or MEG data (Hunt et al., 2012). We have used dynamic causal modelling in this setting to provide a biologically realistic neuronal model of distributed responses that allows us to understand neuronal responses in terms of specific synaptic mechanisms.

Our study complements and extends previous studies of decision-making in humans. Hunt et al. derived similar predictions to those used here, and applied them to MEG data collected during an economic decision-making task, suggesting candidate regions in the ventromedial prefrontal cortex and inferior parietal lobule (Hunt et al., 2012). Recent studies of perceptual decision-making have found evidence for evolving decision signals in centro-parietal EEG electrodes (O'Connell et al., 2012; Kelly and O'Connell, 2013) and MEG sensors (de Lange et al., 2010). It has also been shown that gamma band activity in the visual cortex reflects the encoding of sensory evidence (Siegel et al., 2007), and that the integral of this reflected in lateralised beta band activity reflecting action selection (Donner et al., 2009; Siegel et al., 2011). This fits with predictive coding models, where prediction errors have been associated with fast gamma activity in supragranular layers (Bastos et al., 2012). Elegant trial by trial analysis has also recently suggested a role for low frequency oscillations in modulating the rate of evidence accumulation during perceptual decision-making (Wyart et al., 2012). It has also been shown that beta-band activity in fronto-parietal areas reflects decision accuracy (Donner et al., 2007), and correlates of task difficulty have been found in evoked response potentials (Philiastides and Sajda, 2006; Philiastides et al., 2006). A number of fMRI studies have also suggested a role for the parietal cortex (Tosoni et al., 2008; Liu and Pleskac, 2011) and other areas (Heekeren et al., 2004, 2006; Ho et al., 2009; Philiastides et al., 2011) in evidence accumulation.

One limitation of using dynamic causal modelling for evoked responses is that we are unable to detail responses that are not evoked by an input. As such, we are unable to model neuronal activity immediately prior to response execution, which is an important aspect of evidence accumulation (Gold and Shadlen, 2007). However, given recent demonstrations of similar dynamics in human EEG (O'Connell et al., 2012; Kelly and O'Connell, 2013), together with the fact that the activity we observed resembles slow ramping activity found in non-human primates (Roitman and Shadlen, 2002; Gold and Shadlen, 2007) – both in anatomical location and time course – we assume that the activity we observed reflects evidence accumulation.

In addition to PPC, our source localisation identified regions of the visual cortex and the middle temporal area. A large body of work has implicated MT in motion processing and shown that is has a causal role in decision-making (Britten et al., 1992, 1993; Ditterich et al., 2003). Our study is the first to directly examine effective connectivity between MT and the PPC during perceptual decision-making. In our modelling, the placement of MT below PPC in the winning model fits well with the notion that evolving decision signals in PPC are based on sensory evidence received from MT during the random-dot motion task (Ditterich et al., 2003; Hanks et al., 2006). In a random-dot motion paradigm, the quanta of sensory evidence themselves are not degraded (in the sense that each individual dot as the same spatial precision at each coherence level). Instead, manipulating coherence alters the precision of information about overall motion direction, a high order attribute or cause of visual input. This suggests that variations in precision at the level of PPC (presumably encoding hypotheses about motion direction), rather than lower in the cortical hierarchy are likely to be key. Nonetheless, it is conceivable that precision changes throughout the cortical hierarchy also play a role. In light of the fact that we do not find significant differences between the strength of modulation across different regions in our group level analyses, we make no specific claims about which regions within the network are key for precision-mediated alterations in slow ramping PPC activity and behavioural responding. We acknowledge that this remains an open question for future research.

We did not observe activity in other areas previously implicated in perceptual decision-making such as the dorsolateral prefrontal cortex (Kim and Shadlen, 1999; Philiastides et al., 2011) and frontal eye fields (Gold and Shadlen, 2000). Since we chose to consider only an early phase of the decision-process, it is possible that frontal areas are more closely linked to the executive aspects of decision-making (Gold and Shadlen, 2000, 2007) and may not have been strongly active within the time window we analysed. Our modelling does not imply that only the cortical sources we consider here are important for perceptual decision-making, but rather makes claims about precision and neuronal dynamics, within the network of regions highlighted by source localisation.

Given its putative position within the dorsal stream, and hypothesised role in linking sensory information and motor planning (Gold and Shadlen, 2000; Cisek and Kalaska, 2010), modulation of precision in the PPC (and possibly elsewhere) is an obvious candidate mechanism for a top-down modulatory influence on perceptual decisions (Shipp et al., 2013). This might include effects of prior probabilities and reward contingencies, perhaps driven by the pulvinar, prefrontal cortex or basal ganglia. Exploring the mechanisms by which such top-down factors influence perceptual decision-making represents an important area for future study.

Because we were primarily interested in the effects of estimated subjective coherence (precision) on each trial, we chose to divide up our data by these subjects' response speed rather than objective stimulus features such as coherence levels. Given that there is a strong correlation between response speed and coherence levels in our paradigm (Fig. 1), one can make similar predictions about the relationship between coherence and neuronal responses, though the relationship is likely to be weaker, since response times depend directly upon the dynamics of the network. It is likely that there are multiple sources of response variability over and above differences in the strength of incoming sensory evidence, for example speed–accuracy tradeoffs or local variations in the decision threshold. Indeed, this possibility is suggested by our results when controlling for the effects of motion coherence. To the extent that these additional factors impact on the weighting of sensory evidence they are captured by our model. To the extent that they are mediated by other mechanisms that are not reflected in early evoked responses (for example strength of belief needed to commit to a decision), they simply add noise to our results rather than presenting a confound.

The extent to which ramping activity in the PPC reflects a link between perceptual processing and motor planning, as compared with ‘pure’ evidence accumulation, remains controversial (Gold and Shadlen, 2007; Cisek and Kalaska, 2010; O'Connell et al., 2012). As such, it is possible that a component of the activity we observed (perhaps particularly in the fastest response bin) reflects motor processes as well as sensory ones. Given the significant gap between the end of the time window we modelled (500 ms) and the mean RT associated with the fastest response bin (1068 ms) any motor planning processes will be closely (or even inextricably (Cisek and Kalaska, 2010; Friston et al., 2012)) bound up with the accumulation of sensory evidence, but the imperatives of sensorimotor processing remain a *caveat* when interpreting these results.

In conclusion, our results provide the first direct evidence for slowly evolving decision processes in the human PPC during perceptual decision-making that are akin to those observed in the macaque, and suggest that the dynamics of evidence accumulation in this region is modulated by estimated precision encoded by the gain on pyramidal cells, as predicted by generalised predictive coding (Friston and Kiebel, 2009; Bastos et al., 2012). In addition, we recover the causal structure of an a priori plausible network of regions involved in decision-making about motion. Understanding the core processes involved in decision-making is crucial for any wider account of normal or pathological decisions (Montague et al., 2012), and slow ramping activity in the PPC is likely to be as important in human choice as it is for macaques (Hanks et al., 2006; Gold and Shadlen, 2007). Our results thus represent a step towards understanding the dynamics of decisions in the human brain within the broader context of predictive coding, and bridging the gap that currently exists between our understanding of decision-making in humans, and other primates.
